# A Hybrid Metaheuristic DE/CS Algorithm for UCAV Three-Dimension Path Planning

**DOI:** 10.1100/2012/583973

**Published:** 2012-10-21

**Authors:** Gaige Wang, Lihong Guo, Hong Duan, Heqi Wang, Luo Liu, Mingzhen Shao

**Affiliations:** ^1^Changchun Institute of Optics, Fine Mechanics and Physics, Chinese Academy of Sciences, Changchun 130033, China; ^2^Graduate School of Chinese Academy of Sciences, Beijing 100039, China; ^3^School of Computer Science and Information Technology, Northeast Normal University, Changchun 130117, China

## Abstract

Three-dimension path planning for uninhabited combat air vehicle (UCAV) is a complicated high-dimension optimization problem, which primarily centralizes on optimizing the flight route considering the different kinds of constrains under complicated battle field environments. A new hybrid metaheuristic differential evolution (DE) and cuckoo search (CS) algorithm is proposed to solve the UCAV three-dimension path planning problem. DE is applied to optimize the process of selecting cuckoos of the improved CS model during the process of cuckoo updating in nest. The cuckoos can act as an agent in searching the optimal UCAV path. And then, the UCAV can find the safe path by connecting the chosen nodes of the coordinates while avoiding the threat areas and costing minimum fuel. This new approach can accelerate the global convergence speed while preserving the strong robustness of the basic CS. The realization procedure for this hybrid metaheuristic approach DE/CS is also presented. In order to make the optimized UCAV path more feasible, the B-Spline curve is adopted for smoothing the path. To prove the performance of this proposed hybrid metaheuristic method, it is compared with basic CS algorithm. The experiment shows that the proposed approach is more effective and feasible in UCAV three-dimension path planning than the basic CS model.

## 1. Introduction

Unmanned combat aerial vehicles (UAVs) are remotely piloted or self-piloted aircrafts that can carry many different types of accessories such as cameras, sensors, and communications equipment. They have a very wide range of applications that include both civil and military areas. Some important features that make them very popular are their low cost, smaller size, and their extended maneuver capability because of absence of a human pilot [1]. In particular, UCAV is one of the inevitable trends of the modern aerial weapon equipment, which develop in the direction of unmanned attendance and intelligence. Research on UCAV directly affects battle effectiveness of the air force and is a fundamental and significant research related to safeness of a nation. Trajectory generation and path planning is one of the key technologies in cooperative UCAV combatting. The flight path planning in a large mission area is a typical large-scale optimization problem, a series of algorithms have been proposed to solve this complicated multiconstrained optimization problem, such as differential evolution [2], genetic algorithm [3], ant colony algorithm [4] and its variant [5, 6], chaotic artificial bee colony [7], and intelligent water drops optimization [8]. However, those methods can hardly solve the contradiction between the global optimization and excessive information.

In 1995, Storn and Price firstly proposed a novel evolutionary algorithm (EA): differential evolution (DE) [9, 10], which is a new heuristic approach for minimizing possibly nonlinear and nondifferentiable continuous space functions. It converges faster and with more certainty than many other acclaimed global population-based optimization methods [11]. This new method requires few control parameters, which makes DE more robust, easy to implement, and lends itself very well to parallel computation.

Cuckoo search (CS) is an optimization algorithm developed by Yang and Deb in 2009 [12, 13], which was inspired by the obligate brood parasitism of some cuckoo species by laying their eggs in the nests of other host birds (of other species) [14]. Each egg in a nest represents a solution, and a cuckoo egg represents a new solution. The aim is to use the new and potentially better solutions (cuckoos) to take the place of a not-so-good solution in the nests. In the simplest form, each nest has one egg. An important advantage of CS algorithm is its simplicity. In principle, comparing with other population-based metaheuristic algorithms such as particle swarm optimization and harmony search, there is essentially only a single parameter *p*_*a*_ in CS (apart from the population size). Therefore, it is very easy to implement [15].

However, in the field of UCAV path planning, no application of CS algorithm exists yet. In this work, the Differential Evolution (DE) algorithm is combined with CS algorithm, which uses the DE mutation and crossover operator instead of Lévy flights to form the new cuckoo egg updating strategy, in order to reduce the number of exact evaluations of candidate solutions. The candidate paths are modeled in the physical space and evaluated with respect to the task space. A smooth path is essential for a real UCAV, because nonsmooth path cannot satisfy the turning constraint. In the UCAV community, most researchers apply the Dubins algorithm to generate a smooth path [16]. In this paper, to improve the quality of the paths, we used a computationally efficient path-smoothing method called B-Spline curve smoothing strategy [17]. B-Spline curve is used for path line modeling, and complicated paths can be produced with a small number of control variables. To verify the feasibility and effectiveness of our proposed approach, the series experiments conducted under complicated combating environment demonstrate that our hybrid metaheuristic approach with B-Spline curve path smoothing can generate a feasible optimal three-dimension path of UCAV more quickly than the basic CS algorithm.

The remainder of this paper is structured as follows.  Section 2 describes the mathematical model in UCAV three-dimension path planning problem. In Section 3, preliminary knowledge of DE and CS algorithm is introduced. Then, an improved CS algorithm for UCAV three-dimension path planning is presented in Section 4 and the detailed implementation procedure is also described. Subsequently, a B-Spline curve method for UCAV path smoothing is described in Section 5. The simulation experiments are conducted in Section 6. Finally, Section 7 concludes the paper and discusses the future path of our work.

## 2. Mathematical Model in UCAV Three-Dimension Path Planning 

As a key component of mission planning system [18], path planning for UCAV is the design of optimal flight route to meet certain performance requirements according to the special mission objective and is modeled by the constraints of the terrain, data, threat information, fuel, and time. The goal for three-dimension path planning is to calculate the optimal or near-optimal flight route for UCAV within the appropriate time, which enables the UCAV to break through the enemy threat environments and self-survive with the perfect completion of mission. In our work, we use the mathematical model for UCAV 3-dimension path planning described as follows [5].

**Figure 1:**
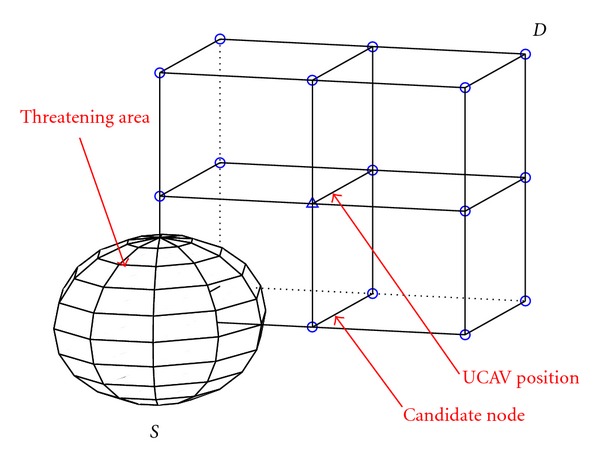
Typical UCAV battle field model in three-dimension.

In order to simplify the UCAV three-dimension path planning problem, the UCAV task region can be divided into three-dimensional mesh, thus forming a three-dimensional network diagram connecting the starting point and end point. In this way, the problem of UCAV optimal three-dimension path planning is the general path optimization problem essentially. The typical UCAV battle field model in three-dimension can be shown in Figure 1.

In Figure 1, suppose the flight task for UCAV is from node *S* to node *D*. There are some threatening areas in the task region. We divide the space into *m* subcubes equally, so there are *n* nodes in the area, which can be labeled with *L*_1_, *L*_2_,…, *L*_*n*_. Let *L*_*i*_(*x*_*i*_, *y*_*i*_, *z*_*i*_) be the *i*th node. It is obvious that there are 26 candidate nodes which could be chosen at most by the UCAV in each step. The nodes in the vertical direction of current point are unaccepted, so the number of the candidate nodes decreases to 24. Then, all the selected nodes could be connected one by one as the step going on until getting the target. In this way, the path from the starting node to the end node can be described as follows:

(1) Path={S,L1(x1,y1,z1),L2(x2,y2,z2),…, Lm−1(xm−1,ym−1,zm−1),D}.

A performance indicator of three-dimension path planning for UCAV mainly contains the completion of the mandatory safety performance indicator, fuel performance indicator, and height performance indicator, that is, indicators with the least threat, the least fuel, and optimal height.

Minimum of performance indicator for threat

(2) $$\begin{array}{c} \min J_{t}=\int_{0}^{L}w_{t}\text{d}l,\;L\;\;\text{is}\;\;\text{the}\;\;\text{length}\;\;\text{of}\;\;\text{the}\;\;\text{path}. \end{array}$$

Minimum of performance indicator for fuel

(3) $$\begin{array}{c} \min J_{f}=\int_{0}^{L}w_{f}\text{d}l,\;L\;\;\text{is}\;\;\text{the}\;\;\text{length}\;\;\text{of}\;\;\text{the}\;\;\text{path}. \end{array}$$

Minimum of performance indicator for height

(4) $$\begin{array}{c} \min J_{h}=\int_{0}^{L}w_{h}\text{d}l,\;L\;\;\text{is}\;\;\text{the}\;\;\text{length}\;\;\text{of}\;\;\text{the}\;\;\text{path}. \end{array}$$

Then the total performance indicators for UCAV route

(5) $$\begin{array}{c} \min J=k_{1}J_{t}+k_{2}J_{f}+\left(1-k_{1}-k_{2}\right)J_{h}\;\left(0\leq k_{1},k_{2}\leq 1\right), \end{array}$$

where *w*_*t*_, *w*_*f*_, and *w*_*h*_ are the threat cost, fuel cost, and height cost for each point on the path that depend on path length, respectively. The choice of *k*_1_ and *k*_2_ all between 0 and 1 gives the designer certain flexibility to dispose relations among the threat exposition degree, the fuel consumption, and the height information. When *k*_1_ is more approaching 1, more attention is paid to the radar's exposed threat, and it requires avoiding the threat as far as possible at the sacrifice of the trajectory length and flight height. Similarly, when *k*_2_ is more approaching 1, a shorter path is needed to be planned regardless of the cost of other two factors.

When the UCAV is flying along the subpath *L*_*ij*_, the total threat cost generated by *N*_*t*_ threats is calculated as follows:

(6) $$\begin{array}{c} w_{t,L_{ij}}=\int_{0}^{L_{ij}}\sum_{k=1}^{N_{t}}\frac{t_{k}}{{\left[{\left(x-x_{k}\right)}^{2}+{\left(y-y_{k}\right)}^{2}\right]}^{2}}\text{d}l. \end{array}$$

**Figure 2:**
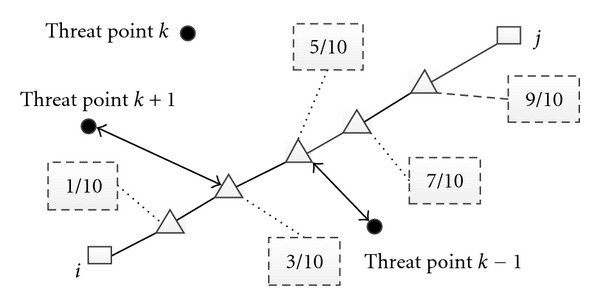
Modeling of the UCAV threat cost [5].

A computationally more efficient and acceptably accurate approximation to the exact solution is to calculate the threat cost at several locations along an edge and take the length of the edge into account. In this work, the threat cost was calculated at five points along each edge, as shown in Figure 2. To simplify the calculations, each path segment is discretized into five subsegments and the threat cost is calculated on the end of each subsegment. If the distance from the threat point to the end of each subsegment is within threat radius, we can calculate the responding threat cost according to

(7) $$\begin{array}{c} w_{t,L_{ij}}=\frac{L_{{\;}_{ij}}^{5}}{5}\sum_{k=1}^{N_{t}}t_{k}\left(\frac{1}{d_{0.1,k}^{4}}+\frac{1}{d_{0.3,k}^{4}}+\frac{1}{d_{0.5,k}^{4}}+\frac{1}{d_{0.7,k}^{4}}+\frac{1}{d_{0.9,k}^{4}}\right), \end{array}$$

where *L*_*ij*_ is the length of the subsegment connecting node *i* and node*j*; *d*_0.1,*k*_ is the distance from the 1/10 point on the subsegment *L*_*ij*_ to the *k*th threat; *t*_*k*_ is threat level of the *k*th threat. Moreover, we can simply consider the fuel cost *w*_*f*_ to *L*, and height cost *w*_*h*,*i*_ equals to *H* which is the flight height of the UCAV when the speed is a constant. The total cost for traveling along the path comes from a weighted sum of the threat and fuel costs shown as in (5).

## 3. Preliminary Knowledge 

### 3.1. Differential Evolution

The differential evolution (DE) algorithm, proposed by Storn and Price [9, 10], is a simple evolutionary algorithm (EA), which generates new candidate solutions by combining the parent individual and a few other individuals of the same population. A candidate substitutes the parent only if it has better fitness. This is a rather greedy selection scheme, which often overtakes traditional EAs. Advantages of DE are easy implementation, simple structure, speed, and robustness. Due to these advantages, it has many real-world applications, such as power dispatch, parameters estimation, economic emission load dispatch, and neural network training.

The mainframe of the original DE algorithm is described in Algorithm 1, where*D* is the number of decision variables. NP is the size of the parent population *P*. *F* is the mutation scaling factor. CR is a constant for crossover operator. *X*_*i*_(*j*) is the *j*th variable of the solution *X*_*i*_. *U*_*i*_ is the offspring. ⌈NP∗rand⌉ is a uniformly distributed random integer number between 1 and NP. And rand is a uniformly distributed random real number in interval (0, 1). Different types of strategies of DE have been proposed depending on the target vector selected and the number of difference vectors used. We use the DE/rand/1/bin scheme shown in Algorithm 1. From Algorithm 1, we can see that there are only three control variables in this algorithm, which are NP, *F*, and CR.

### 3.2. Cuckoo Search (CS)

Cuckoo has a smart reproduction strategy that involves the female laying her fertilized eggs in the nest of another species so that the replaced parents unwittingly raise her brood. Sometimes the cuckoo's eggs in the nest are discovered and the surrogate parents throw them out or leave the nest and start their own brood elsewhere [14].

Cuckoo search (CS) is a new metaheuristic algorithm for solving optimization problems, which is based on the obligate brood parasitic behavior of some cuckoo species in combination with the Lévy flight behavior of some birds and fruit flies. In the case of CS, the walking steps of a cuckoo are determined by the Lévy flights.

A Lévy flight is a random walk in which the steps are defined in terms of the step-lengths, which have a certain probability distribution, with the directions of the steps being isotropic and random. Lévy flights is a class of random walk in which the jumps are distributed according to a power law, that is,

(8) $$\begin{array}{c} y=x^{-\beta }, \end{array}$$

where 1 < *β* < 3 and therefore has an infinite variance. 

Barthelemy et al. [19] had reported the relationship between light, and Lévy flights has subsequently been applied to improve and optimize searching. In the case of CS, the walking steps of a cuckoo are determined by the Lévy flights.

For simplicity in describing cuckoo search in [12], Yang and Deb used the following three idealized rules.

1. Each cuckoo lays only one egg at a time, and places its egg in a selected nest at random.
2. The best nests with high quality of eggs will carry over to the next generation.
3. The number of available host nests is fixed, and the egg laid by a cuckoo is discovered by the host bird with a probability *p*_*a*_ ∈ [0, 1]. In this case, the host bird can either throw the egg away or leave the nest, and build a fully new nest. For simplicity, this last assumption can be approximated by the fraction *p*_*a*_ of the *n* nests which are displaced by new nests (with new random solutions) [15].

Based on these three rules, the basic steps of the CS can be summarized as shown in Algorithm 2. 

In CS, each egg in a nest represents a solution, and a cuckoo egg represents a new solution. The aim is to use the new and potentially better solutions (cuckoos) to replace a not-so-good solution in the nest. In the simplest form, each nest has one egg. The algorithm can be extended to more complicated cases in which each nest has multiple eggs representing a set of solutions.

When generating new solutions *x* ^(*t*+1)^ for, say, a cuckoo *i*, a Lévy flight is performed

(9) $$\begin{array}{c} x_{i}^{\left(t+1\right)}=x_{i}^{\left(t\right)}+\alpha ⊕\text{L}\hat{\text{e}}\text{vy}\left(\lambda \right), \end{array}$$

where *α* > 0 is the step size which should be related to the scales of the problem of interests. In most cases, we can use *α* = 1. The above equation is essentially the stochastic equation for random walk. In general, a random walk is a Markov chain whose next status/location only depends on the current location (the first term in the above equation) and the transition probability (the second term). The product ⊕ means entrywise multiplications. This entrywise product is similar to those used in PSO, but here the random walk via Lévy flight is more efficient in exploring the search space as its step length is much longer in the long run.

The Lévy flights essentially provides a random walk, while the random step length is drawn from a Lévy distribution

(10) $$\begin{array}{c} \text{L}\hat{\text{e}}\text{vy}\left(\lambda \right)\sim u=t^{-\lambda }\;\left(1<\lambda \leq 3\right), \end{array}$$

which has an infinite variance with an infinite mean. Here the steps essentially form a random walk process with a power-law step-length distribution with a heavy tail. Some of the new solutions should be generated by Lévy walk around the best solution obtained so far; this will speed up the local search. However, a substantial fraction of the new solutions should be generated by far field randomization and whose locations should be far enough from the current best solution; this will make sure the system will not be trapped into a local optimum.

## 4. Differential Evolution/Cuckoo Search: DE/CS

Generally speaking, the standard DE algorithm is adept at exploring the search space and locating the region of global optimal value, but it is not relatively good at exploiting solution. On the other hand, standard CS algorithm is usually quick at the exploitation of the solution though its exploration ability is relatively poor. Therefore, in this paper, a hybrid metaheuristic algorithm by integrating differential evolution into cuckoo search, so-called DE/CS, is used to solve the three-dimension path planning for UCAV. The difference between DE/CS and CS is that the mutation and crossover of DE is used to replace the original CS selecting a cuckoo. In this way, this method can explore the new search space by the mutation of the DE algorithm and exploit the population information with CS and therefore can conquer the lack of the exploitation of the DE algorithm. In the following, we will show the algorithm DE/CS, which is a variety of DE and CS.

### 4.1. Mainframe of DE/CS

The critical operator of DE/CS is the hybrid differential evolution selecting cuckoo operator, which embeds the differential evolution into the CS. The core idea of the proposed differential evolution selecting cuckoo operator is based on two considerations. First, the mutation operator of DE can add diversity of the population to improve the search efficiency. Second, the mutation operator of DE can improve the exploration of the new search space. Pseudocode of hybrid differential evolution selecting cuckoo operator can be described as in Algorithm 3. In Algorithm 3, *D* is the number of decision variables. NP is the size of the parent population *P*. *F* is the mutation scaling factor. CR is a constant for crossover operator. *X*_*i*_(*j*) is the *j*th variable of the candidate solution *X*_*i*_. *X*_*u*_ is the offspring. ⌈NP∗rand⌉ is a uniformly distributed random integer number between 1 and NP. And rand is a uniformly distributed random real number in interval (0, 1). We use the DE/rand/1/bin scheme shown in Algorithm 3.

By incorporating above-mentioned hybrid differential evolution selecting cuckoo operator into original CS algorithm, the DE/CS has been developed as a new algorithm. DE/CS algorithm is given as in Algorithm 4, where a fraction of worse nests are discovered with a probability *p*_*a*_.*K* is a status matrix with NP × *D* whose value is logical value 0 or 1, meaning the egg in the nest discovered or not, and *K*(*i*, :) represents the *i*th row elements in the status matrix *K*. The Hadamard product of two matrices *μ*⊙*υ* is defined as the entrywise product, that is, [*μ*⊙*υ*] = *μ*_*ij*_ *υ*_*ij*_. In the real world, if a cuckoo's egg is very similar to host's eggs, then this cuckoo's egg is less likely to be discovered, thus the fitness should be related to the difference in solutions. Therefore, it is a good idea to do a random walk in a biased way with some random step sizes. Vector *Step* is the step size that determines how far a random walker can go for a fixed number of iterations. *P*_1_ and *P*_2_ are the copy of the population *P*; *Y*_*i*_ and *Z*_*i*_ are the individuals in the population *P*_1_ and *P*_2_, respectively. From Algorithm 4, we can see that there are only four control parameters in this algorithm, which are NP, *F*, CR, and *p*_*a*_.

### 4.2. Algorithm DE/CS for UCAV Three-Dimension Path Planning

In essence, UCAV three-dimension path planning is to reach minimum value for the objective function shown as in (5). For a minimization problem, the quality or fitness of a solution can simply be inversely proportional to the value of the cost function (5). For simplicity, we can use the following simple representations that each egg in a nest represents a solution, and a cuckoo egg represents a new solution; the aim is to use the new and potentially better solutions (cuckoos) to replace a not-so-good solution in the nests. For this present work, we will use the simplest approach where each nest has only a single egg. In this case, there is no distinction between egg, nest, or cuckoo, as each nest corresponds to one egg which also represents one cuckoo. Therefore, in the following, we do not distinguish the egg, nest, and cuckoo all of which represent a candidate solution.

Let NP cuckoos be in the starting node; the cuckoos will choose the next nodes in the grid network diagram according to the selecting cuckoo rule shown as in Algorithm 3 instead of the Lévy flights used in CS shown in (8). A cuckoo lays an egg in a nest which may be found by the hosting bird; if then, the egg would be discarded, and then it is replaced by another cuckoo's egg. Thus cuckoo birds are always looking for a better place in order to decrease the chance of their eggs to be discovered. The process can be approximated by the fraction *p*_*a*_ of the NP nests which are displaced by new nests (with new random solutions). Consequently, it will enhance the original quality of the candidate solution. Thus, the more cuckoos a UCAV path is passed by, the bigger possibility that a path can be selected by the other cuckoos. This process can guarantee nearly all cuckoos walk along the shortest UCAV path in the end.

Based on the above analysis, the pseudocode of improved CS-DE/CS for UCAV three-dimension path planning is described as follows (Algorithm 5).

## 5. Path-Smoothing Strategies

The generated UCAV optimal three-dimension path using the proposed hybrid metaheuristic method DE/CS is usually hard for exact flying. There are some turning points on the optimized path [20, 21]. In this section, we adopt a class of dynamically feasible trajectory smooth strategy called B-Spline curves smoothing strategy [17]. B-Splines are adopted to define the UCAV desired path, providing at least first-order derivative continuity. B-Spline curves are well fitted in the evolutionary procedure; they need a few variables (the coordinates of their control points) in order to define complicated curved paths. Each control point has a very local effect on the curve's shape and small perturbations in its position produce changes in the curve only in the neighborhood of the repositioned control point. 

B-Spline curves are parametric curves, with their construction based on blending functions [22]. Their parametric construction provides the ability to produce nonmonotonic curves. If the number of control points of the corresponding curve is *n* + 1, with coordinates *w*_0_(*x*_0_, *y*_0_, *z*_0_),…, *w*_*n*_(*x*_*n*_, *y*_*n*_, *z*_*n*_), the coordinates of the B-Spline curve may be written as

(11) $$\begin{array}{c} x\left(u\right)=\sum_{i=1}^{n}x_{i}\cdot N_{i,p}\left(u\right),\\ y\left(u\right)=\sum_{i=1}^{n}y_{i}\cdot N_{i,p}\left(u\right),\\ z\left(u\right)=\sum_{i=1}^{n}z_{i}\cdot N_{i,p}\left(u\right), \end{array}$$

where *u* is the free parameter of the curve, *N*_*i*,*p*_(*u*) are the blending functions of the curve, and *p* is its degree, which is associated with curve's smoothness (*p* + 1 being its order). Higher values of *p* correspond to smoother curves. 

The blending functions are defined recursively in terms of a *knot* vector *U* = {*u*_0_,…, *u*_*m*_}, which is a nondecreasing sequence of real numbers, with the most common form being the *uniform nonperiodic* one, defined as

(12) $$\begin{array}{c} u_{i}=\{\begin{array}{cc} 0 & \text{if}\;\;i<p+1,\\ i-p & \text{if}\;\;p+1\leq i\leq n,\\ n-p+1 & \text{if}\;\;n<i. \end{array} \end{array}$$

The blending functions *N*_*i*,*p*_ are computed, using the knot values defined above, as

(13) $$\begin{array}{c} N_{i,0}=\{\begin{array}{cc} 1 & u_{i}\leq u\leq u_{i+1},\\ 0 & \text{otherwise}, \end{array}\\ N_{i,p}\left(u\right)=\frac{u-u_{i}}{u_{i+p}-u_{i}}N_{i,p-1}\left(u\right)+\frac{u_{i+p+1}-u}{u_{i+p+1}-u_{i+1}}N_{i+1,p-1}\left(u\right). \end{array}$$

If the denominator of either of the fractions is zero, that fraction is defined to have zero value. Parameter *u* varies between 0 and (*n* − *p* + 1) with a constant step, providing the discrete points of the B-Spline curve. The sum of the values of the blending functions for any value of *u* is always 1. 

**Figure 3:**
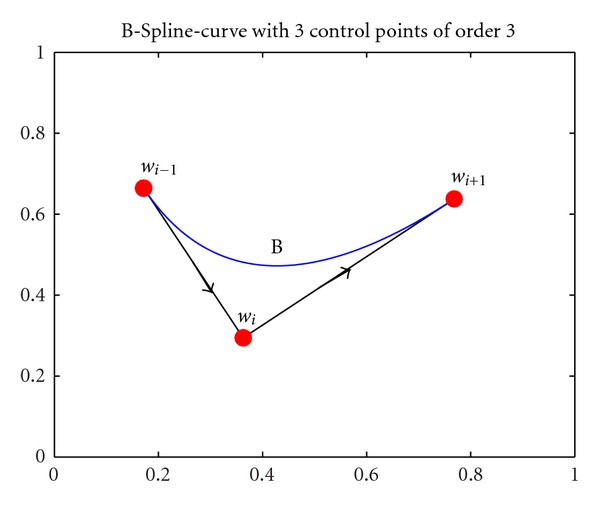
A quadratic (*p* = 2) 2-dimensional B-Spline curve, produced using a uniform nonperiodic knot vector, and its control polygon.

The use of B-Spline curves for the determination of a flight path provides the advantage of describing complicated nonmonotonic 3-dimensional curves with controlled smoothness with a small number of design parameters, that is, the coordinates of the control points. Another valuable characteristic of the adopted B-Spline curves is that the curve is tangential to the control polygon at the starting and end points. This characteristic can be used in order to define the starting or end direction of the curve, by inserting an extra fixed point after the starting one, or before the end control point.  Figure 3 shows a quadratic 2-dimensional B-Spline curve (*p* = 2) with its control points and the corresponding control polygon.

After this process, the original path $\bar{w_{i-1}w_{i}}\to \bar{w_{i}w_{i+1}}$ could be replaced by the path $\hat{w_{i-1}B}\to \hat{Bw_{i+1}}$. In this way, the optimized path can be smoothed for feasible flying. This trajectory smoothing algorithm has a small computational load and can be run in real time.

## 6. Simulation Experiments

In this section, we look at the performance of the proposed hybrid metaheuristic DE and CS to UCAV three-dimension path planning through a series of experiments conducted under complex combat field environment. 

To allow a fair comparison of running times, all the experiments were implemented on a PC with a Pentium IV processor running at 2.0 GHz, 512 MB of RAM, and a hard drive of 160 Gbytes. Our implementation was compiled using MATLAB R2012a (7.14) running under Windows XP3. No commercial CS tools or other population-based optimization tools were used in the following experiments. 

To our knowledge, parameter setting has a great effect on the performance of optimization method. According to simulation experiments [12], Yang and Deb found that population size NP = 15 to 40 and discovery rate *p*_*a*_ = 0.25 are sufficient for most optimization problems. Their results and analysis also illustrate that the convergence rate, to some degree, is insensitive to the parameters selected. This means that we do not need to fine-tune parameters for any given problems. Therefore, in all experiments, we will use the same set of CS algorithm parameter, which are step size *α* = 1, discovery rate *p*_*a*_ = 0.25, population size NP = 30, and maximum generation Maxgen = 200.

**Figure 4:**
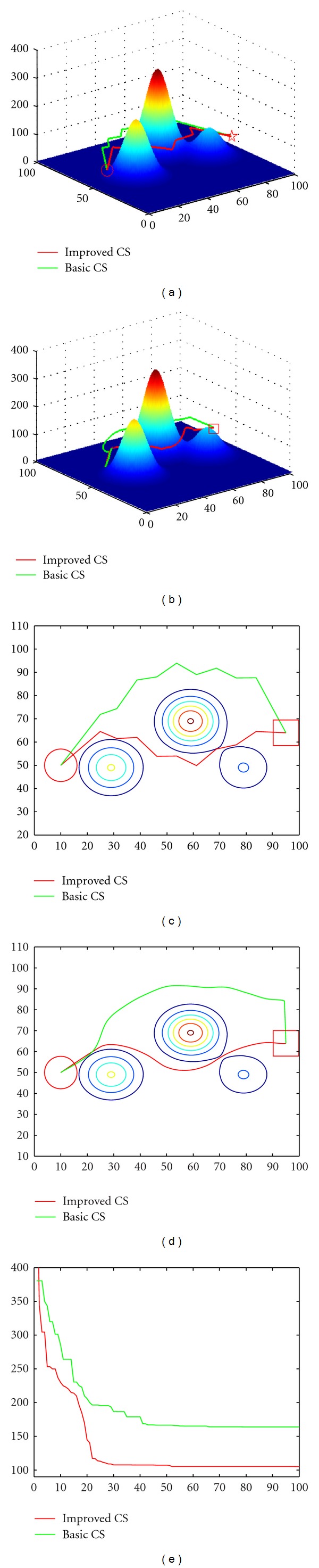
Parameter values were NP = 30, *p*_*a*_ = 0.25. (a) Path-planning original results comparison between basic CS and improved CS in a three-dimension space. (b) Route comparison after using the smoothing strategy in a three-dimension space. (c) Path-planning original results comparison between basic CS and improved CS in a two-dimension space. (d) Route comparison after using the smoothing strategy in a two-dimension space. (e) Evolution curves comparison between the basic CS and the improved CS.

Figure 4 shows the UCAV path planning results comparison between basic CS and the proposed hybrid metaheuristic CS and DE algorithm in three-dimension and two-dimension space with NP = 30, *p*_*a*_ = 0.25, and the curve path comparison by the smooth algorithm, and also the evolution curves comparison. The symbol “○” denotes the starting point, the cone denotes the threaten area, while the symbol “□” denotes the end point. And the green line is the path generated by the basic CS, while the red one is generated by the improved CS. 

The values of each optimal solution searched by the different algorithm could be given by the value of the “shortest length,” which can be shown in Table 1. Table 1 shows the results found by basic CS and improved CS algorithm over 100 Monte Carlo runs. From the experimental results presented in Figure 4 and Table 1, it is apparent that the proposed hybrid metaheuristic CS and DE method can find feasible and optimal three-dimension path for the UCAV very quickly and can effectively solve the three-dimension path planning of UCAV in complicated combating environments. This method provides a new way for three-dimension path planning of UCAV in real application.

## 7. Conclusion and Future Work

This paper presented a hybrid metaheuristic CS and DE algorithm for UCAV three-dimension path planning in complicated combat field environments. A novel type of CS model has been described for single UCAV path planning, and DE is applied to optimize selecting cuckoo operator during the process of egg updating in nest. Then, the UCAV can find the safe path by connecting the chosen nodes while avoiding the threat areas and costing minimum fuel. This new approach can accelerate the global convergence speed while maintaining the strong robustness of the basic CS. The detailed implementation procedure for this metaheuristic approach is also described. In order to make the optimized UCAV path more feasible, the B-Spline curve is adopted for smoothing the path, and this trajectory smoothing algorithm has a small computational load and can be run in real time. Compared with the basic CS algorithm, the simulation experiments show that this method is a feasible and effective way in UCAV path planning. It is also flexible, in complicated dynamic battle field environments, and pop-up threats are easily incorporated. 

In the algorithm of UCAV three-dimension path planning, there are many issues worthy of further study, and efficient route planning method should be developed depending on the analysis of specific combat field environments. Currently, the hot issue contains self-adaptive route planning for a single UCAV and collaborative route planning for a fleet of UCAVs. As the important ways of improving aircraft survivability, adaptive route planning should analyze real-time data under the uncertain and dynamic threat condition, it can even remodify preplanned flight path to improve the success rate of completing mission. The difficulty of the collaborative route planning for a fleet of UCAVs exists in coordination between the various UCAVs, including the fleet formation, target distribution, arrival time constraint, and avoidance conflict, each of which is a complicated question worthy of further study. Our future work will focus on the two hot issues and develop new methods to solve problem in UCAV 3-dimension path planning.

## Figures and Tables

**Algorithm 1:**
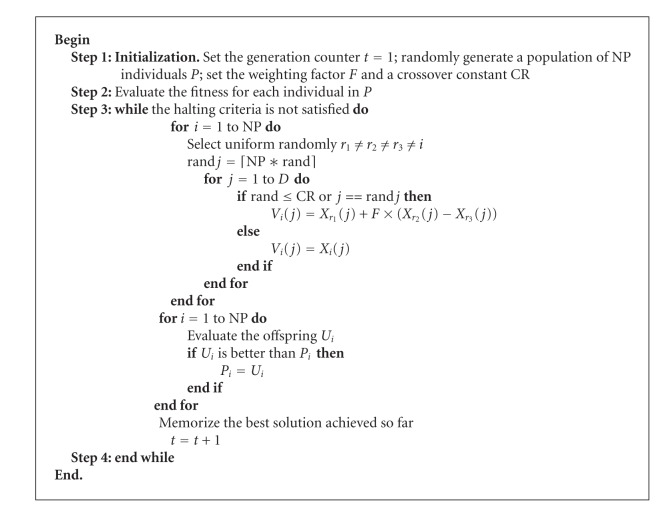
Algorithm of DE with DE/rand/1/bin scheme.

**Algorithm 2:**
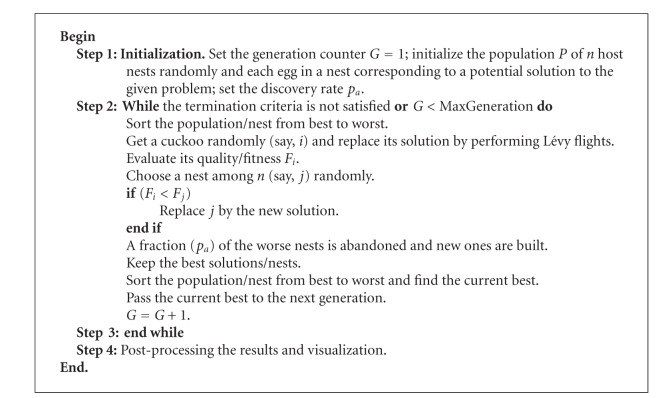
The algorithm of cuckoo search (CS) via Lévy flights.

**Algorithm 3:**
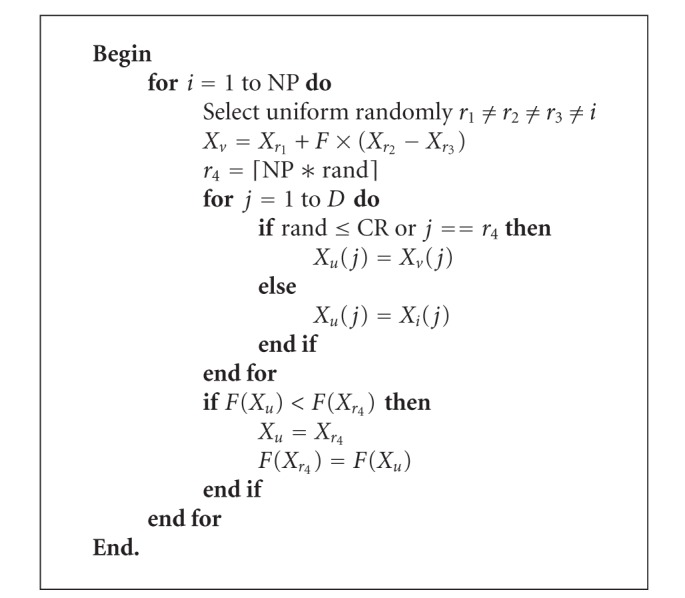
Algorithm of selecting cuckoo for DE/CS.

**Algorithm 4:**
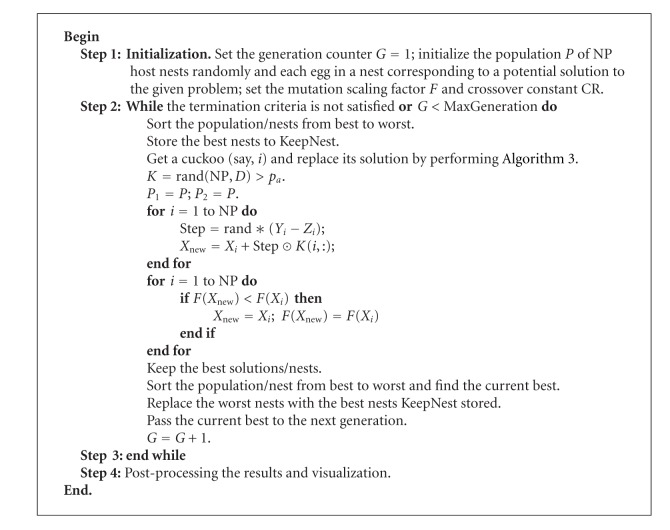
The main procedure of DE/CS.

**Algorithm 5:**
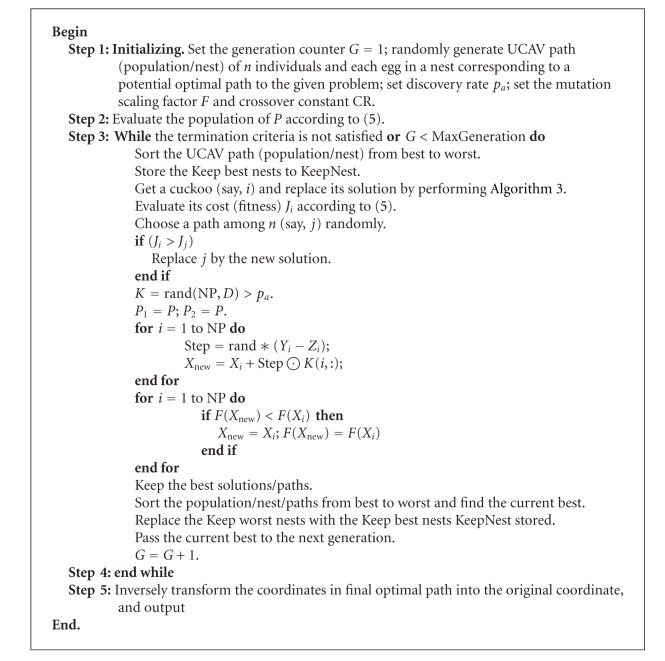
Algorithm of DE/CS for UCAV three-dimension path planning.

**Table 1:** Shortest length comparison between the basic CS and the improved CS in UCAV three-dimension path planning problem. The numbers shown are the results found after 100 Monte Carlo simulations of each algorithm.

	Basic CS	Improved CS
Mean	193.1960	165.5124
Std	34.9554	25.0811
Best	127.1038	105.1268
Worst	282.3626	207.9992
Time (sec)	75.6491	26.1248
